# Immunomodulatory Effects of Diterpene Quinone Derivatives from the Roots of *Horminum pyrenaicum* in Human PBMC

**DOI:** 10.1155/2018/2980295

**Published:** 2018-01-14

**Authors:** K. Becker, S. Schwaiger, B. Waltenberger, D. Fuchs, C. K. Pezzei, H. Schennach, H. Stuppner, J. M. Gostner

**Affiliations:** ^1^Division of Biological Chemistry, Biocenter, Medical University of Innsbruck, Innsbruck, Austria; ^2^Institute of Pharmacy/Pharmacognosy and Center for Molecular Biosciences Innsbruck (CMBI), University of Innsbruck, Innsbruck, Austria; ^3^Institute for Analytical Chemistry and Radiochemistry and CMBI, University of Innsbruck, Innsbruck, Austria; ^4^Central Institute of Blood Transfusion and Immunology, University Hospital of Innsbruck, Innsbruck, Austria; ^5^Division of Medical Biochemistry, Biocenter, Medical University of Innsbruck, Innsbruck, Austria

## Abstract

Several phytochemicals were shown to interfere with redox biology in the human system. Moreover, redox biochemistry is crucially involved in the orchestration of immunological cascades. When screening for immunomodulatory compounds, the two interferon gamma- (IFN-*γ*-) dependent immunometabolic pathways of tryptophan breakdown via indoleamine 2,3-dioxygenase-1 (IDO-1) and neopterin formation by GTP-cyclohydrolase 1 (GTP-CH-I) represent prominent targets, as IFN-*γ*-related signaling is strongly sensitive to oxidative triggers. Herein, the analysis of these pathway activities in human peripheral mononuclear cells was successfully applied in a bioactivity-guided fractionation strategy to screen for anti-inflammatory substances contained in the root of *Horminum (H.) pyrenaicum* L. (syn. Dragon's mouth), the only representative of the monophyletic genus *Horminum*. Four abietane diterpene quinone derivatives (horminone, 7-*O*-acetylhorminone, inuroyleanol and its 15,16-dehydro-derivative, a novel natural product), two nor-abietane diterpene quinones (agastaquinone and 3-deoxyagastaquinone) and two *abeo* 18 (4 → 3) abietane diterpene quinones (agastol and its 15,16-dehydro-derivative) could be identified. These compounds were able to dose-dependently suppress the above mentioned pathways with different potency. Beside the description of new active compounds, this study demonstrates the feasibility of integrating IDO-1 and GTP-CH-I activity in the search for novel anti-inflammatory compounds, which can then be directed towards a more detailed mode of action analysis.

## 1. Introduction

Throughout the history of mankind, natural product extracts were used for the treatment of a broad range of inflammation-associated symptoms such as infections, fever, or pain [1]. Nowadays, natural products are innovative sources for the development of modern therapeutic drugs [2]. Approximately 25% of the modern medications originate from plants and/or phytochemicals [3]. Still, there is an enormous interest in the identification and analysis of phytochemicals to discover more effective agents for the treatment of diseases or the alleviation of symptoms that are associated with immune activation and inflammation. Specific therapeutic targets can be addressed with bioactivity-guided fractionation strategies, which have emerged as useful tools in the identification of active metabolites from plant extracts and complex mixtures.

Immune activation and oxidative stress is linked with a number of chronic diseases such as infections, autoimmune syndromes, malignancies, neurodegenerative and cardiovascular disorders. The modulation of processes by which oxidative stress induces inflammation and *vice versa* is suggested to be a general property of substances, which have anti-inflammatory, anti-aging, and health promoting effects [4, 5].

Several plants of the Lamiaceae family, which is rich in diterpene compounds, are known for their antioxidant and/or anti-inflammatory activities, including sage, thyme, rosemary, and lavender [6]. After an initial screening, a dichloromethane extract of the roots of *Horminum pyrenaicum* L. (syn.: Pyrenean Dead-nettle or Dragon's mouth), a European representative of the Lamiaceae family, was chosen for bioguided isolation.

Genetic studies positioned the genus *Horminum* within the tribe Mentheae into a close relation to the genera *Prunella* and *Cleonia* [7]. *H. pyrenaicum* is the only representative of this monophyletic genus. It grows on chalky and stony soils as well as on rough pastures in a height around 1400 till 2500 m and is domestic in the south of the Alps and in the Pyrenees [8]. It is a perennial herbaceous plant, which is able to reach 10 to 30 cm height with a strong ligneous tap-root and a plain or weakly branched rhizome [9]. Known constituents of *H. pyrenaicum* are diterpenes like horminone (=7-hydroxyroyleanone) and 7-*O*-acetylhorminone in the aerial plant parts as well as agastaquinone, coleon U 12-methylether and 3-deoxyagastaquinone. So far, the latter compound is only described from this species, and it is contained in the sub-aerial plant parts [10].

Quinones are ubiquitous in nature and derivatives of quinones are common components of biologically relevant molecules such as K vitamins or compounds that are directly involved in oxidative metabolism like coenzyme Q10.

The diterpene derivatives (phytocannabinoids) present in *Cannabis* plants (*Cannabis sativa* L., *Cannabis indica* Lam.) were previously reported to exert potent anti-inflammatory and immunomodulatory effects, which could also be demonstrated in an *in vitro* setting of mitogen-induced peripheral blood mononuclear cells (PBMC), by applying neopterin formation and tryptophan breakdown as sensitive and convenient readouts [11]. This *in vitro* model has been used to monitor immunomodulatory activities for a broad variety of phytochemicals, drugs, or plant extracts [12].

Briefly, PBMC are isolated from whole blood, treated with test substances and stimulated with mitogens such as phytohaemagglutinin (PHA), which efficiently induce the release of interferon-γ (IFN-γ) and initiate T helper (Th) type 1 responses. Consequently, tryptophan breakdown and neopterin formation via the enzymes indoleamine 2,3-dioxygenase-1 (IDO-1) and GTP-cyclohydrolase 1 (GTP-CH-I) is initiated [13, 14]. The measurement of these biomarkers allows evaluation and quantification of the immunomodulatory properties of compounds in an *in vitro* setting, where the interplay between human T-cells and monocytes/macrophages is mimicked.

Activation of the biochemical pathways of tryptophan catabolism and neopterin formation are central events during the cellular (Th1-type) immune reaction [15]. Dysregulation and/or overactivation of these pathways is associated with infections, cardiovascular diseases, neurodegenerative disorders, with many different types of cancers, and with the process of aging [16–19]. The Th1-type cytokine IFN-γ is the major inducer of these and many other pro-inflammatory signaling cascades [20]. IDO-1 catalyzes the rate-limiting step in the conversion of the essential amino acid tryptophan into kynurenine [14, 21]. The kynurenine to tryptophan ratio (Kyn/Trp) is indicative for the activity of IDO-1, when additional inflammation markers such as neopterin are present [22]. Neopterin is formed by the enzyme GTP-CH-I in response to IFN-*γ*, and has been applied as biomarker of Th1-type immune response in several *in vitro* and clinical settings [23]. High Kyn/Trp and high neopterin levels usually coincide in patients suffering from various Th1-related immunopathologies [19].

Within this study, bioactive diterpene quinones derived from *H. pyrenaicum* were identified through a bioactivity-guided separation and isolation process, by focusing on immunomodulatory, preferentially anti-inflammatory properties. The bioactivity screen was based on the test system described above, using mitogen-stimulated human PBMC and tryptophan breakdown as well as neopterin formation, two important immunometabolic pathways of the cellular immune response, as readout.

## 2. Material and Methods

### 2.1. General Experimental Procedures

All reagents used were of analytical grade and were purchased from Sigma Aldrich (Vienna, Austria). HPLC solvents were of gradient grade. Technical grade solvents were distilled before use. Water was produced by reverse osmosis followed by distillation. Optical rotations were measured using a Perkin-Elmer (Wellesley, MA) 341 polarimeter. 1D- and 2D–NMR spectra were acquired with a Bruker (BrukerBiospin, Rheinstetten, Germany) Ultrashield plus 600 spectrometer or a Bruker DRX 300 spectrometer using CDCl_3_ or deuterated acetonitrile (containing 0.03% TMS) (Euriso-Top, Saint-Aubin, France). Chemical shift values were referenced to the residual solvent signals. HPLC analyses were carried out using an HP 1050 system (Agilent, Waldbronn, Germany) equipped with autosampler, DAD, and column thermostat. Separations were performed on a Phenomenex (Torrance, CA) Luna 3 *μ*m Phenyl-Hexyl column (150 × 3.00 mm). A mobile phase consisting of 0.02% TFA and 1% THF in H_2_O (*v/v*) (solvent A) and 25% THF in MeOH (*v/v*) (solvent B) was employed with gradient elution (0 min, 40% B; 5 min, 40% B; 15 min, 65% B; 40 min, 80% B; 50 min, 99% B; 70 min, 99% B). The detection wavelength was 280 nm, and the thermostat was set at 45°C. The injection volume was 10 *μ*L; the flow rate was 0.3 mL/min. For LC-ESI-MS experiments, the HPLC system was coupled to a Bruker (Bruker Daltonics, Bremen, Germany) Esquire 3000plus iontrap, replacing solvent A with a solution of 0.1% acetic acid and 0.9% formic acid in H_2_O (*v/v*). The MS parameters were as follows: splitless; ESI negative and positive mode; spray voltage −4.5 kV; nebulizer gas 30 psi; drying gas flow rate 10.00 L/min; *m/z* range 50–1500; capillary temperature 350°C. For high-speed countercurrent chromatography (HSCCC) separations a P.C. Inc. (Potomac, MD) series 690 multilayer (triple) coil HSCCC instrument with a Gilson (Villiers-le-Bel, France) pump system (model 302/803 C) was used. Semipreparative HPLC separations were carried out on a Dionex (Dionex Softron, Germering, Germany) system fitted with a P580 pump, a ASI-100 autosampler, a UVD 170 U detector, a Gilson 206 fraction collector, and a Phenomenex Synergi Polar-RP 80A column (150 × 4.6 mm i.d., 4 *μ*m). A solvent system of H_2_O (solvent A) and 25% THF in MeOH (solvent B) was used for isocratic elution with 35% A and 65% B. The detection wavelength was 280 nm, the temperature 45°C, the injection volume 25 *μ*L, and the flow rate was 1 mL/min. Sephadex LH-20 (Pharmacia Biotech, Uppsala, Sweden) and silica gel (VWR, Darmstadt, Germany) were used as stationary phases for CC. Thin layer chromatography (TLC) was carried out on silica gel 60 F254 plates (VWR, Darmstadt, Germany) using suitable mobile phases, and detection was performed at UV 254 and 366 as well as after derivatization with vanillin/H_2_SO_4_ (1% *w/v* and 5% *v/v* methanolic solutions, resp.). Preparative TLC was performed using silica gel 60 F254 PLC plates (Merck, Darmstadt, Germany).

### 2.2. Plant Material and Extraction

*H. pyrenaicum* was collected at the southeast of the Sellajoch (1930 m altitude), Italy, at the 22nd of August 2003. A voucher specimen (UA-030822_A1) was deposited at the Herbarium of the Institute of Pharmacy/Pharmacognosy, University of Innsbruck, Austria. The air-dried root material (842 g) was grounded and defatted by exhaustive maceration with petroleum ether at room temperature yielding 4.62 g of red-brown petroleum ether extract (0.55%). For a prescreen for potential biological activity in the cell-based assay system described below, 25.0 g of the defatted plant material was re-extracted by sonication for 15 minutes with 200 mL dichloromethane (DCM). This procedure was repeated three-times, yielding 341 mg after evaporation of the solvent (1.36%). For compound isolation, 750 g of the dried plant material were re-extracted by Soxhlet extraction with dichloromethane for 24 hours at room temperature, yielding 14.1 g of extract.

### 2.3. Fractionation for Activity Localization

The DCM-extract (pretrial, 200 mg) was subjected to fractionation on a Sephadex-LH20 column (Pharmacia Biotech No. 17–0090-01, 2 cm × 70 cm) using a DCM-acetone mixture (85 : 15, *v/v*) as eluent. Eight fractions (HOR-a1 to HOR-a8) were collected for further testing in the cell-based screening system.

### 2.4. Isolation of Diterpene Quinones

10.6 g of the dichloromethane extract was subjected to silica gel column chromatography (Silicagel 60, grain size 40–63 *μ*m, Merck No. 1.09385) using a gradient of hexane to DCM as mobile phase. A part of the fraction (195.2 mg) eluted at 580 mL to 650 mL with hexane/DCM (95 + 5), was used for further separation. The obtained fraction was purified by HSCCC using a system of heptane/MeCN/DCM (10 + 6 + 3, *v/v/v*) using the upper phase as mobile phase (head to tail) with a flow-rate of 1.00 mL/min, 800 rpm, in a 230 mL coil. The eluate was combined to 14 fractions. Fraction 5 (eluted at 270–315 mL) contained 7.8 mg of pure compound 8. Fraction 10 (eluted at 595–680 mL) was purified by preparative TLC using chloroform as mobile phase, yielding 3.6 mg of compound 3. The same procedure was applied to fraction 9 (eluted at 535–590 mL) revealing 4.1 mg of compound 2. Fraction 7 (eluted at 340–415 mL) was further purified by semi-preparative HPLC; 2.0 mg of compound 6 and 3.5 mg of compound 7 could be purified in this way. Fraction 8 (eluted at 420–530 mL) was further purified by Sephadex-LH20 CC using DCM/acetone (85 + 15, *v/v*) as mobile phase yielding 8.9 mg of compound 1 as well as a fraction (13 mg) containing compounds 4 and 5. Due to the small amount of the obtained mixture, the structure elucidation of compounds 4 and 5 was performed by means of LC-SPE-NMR [24]. Therefore, the compound mixture (13.2 mg) was dissolved in 1.00 mL methanol. The solution was injected into the HPLC system (ten times 20 *μ*L) and separated on a Phenomenex Synergy Polar RP 80A column (150 × 4.2 mm, 4 *μ*m) with an isocratic mixture of 40% water and 60% methanol containing 25% THF at 40°C and a flow rate of 0.80 mL/min. The corresponding peaks (compound 4 at 33.5 min, 0.08 mg; compound 5 at 35.9 min, 0.20 mg) were trapped on SPE-cartridges, dried and transferred into a Bruker Avance II 600 NMR system with deuterated acetonitrile.

NMR data of 15,16-dehydroinuroyleanol (4) in deuterated acetonitrile: *δ*_C_ (150.90 MHz, in ppm), δ_H_ (600.13 MHz, in ppm, multiplicity, coupling constants *J* in Hz in brackets): 1: 36.8, 3.31 *td* (6.2/3.4), 1.32 *m,* 2H; 2: 19.4, 1.77 *td* (3.6/13.9), *1.55 dtd* (3.7/7.5/7.8/11.4), 2H; 3: 41.6, 1.48 *m*, 1.30 *m,* 2H; 4: 33.9; 5: 50.3, 1.81 *dd* (2.6/14.8), 1H; 6: 36.1 2.71 *dd* (14.8/17.1), 2.56 *dd* (2.5/17.2), 2H; 7: 207.2; 8: 112.6; 9: 137.7; 10: 40.9; 11: 138.6; 12: 153.2; 13: 123.0; 14: 156.4; 15: 138.5; 16: 118.0, 5.34 *td* (1.7/3.4), 4.95 *br s*, 2H; 17: 23.1, 2.06 *s*, 3H; 18: 32.9, 0.94 *s*, 3H; 19: 21.6, 0.97 *s*, 3H; 20: 17.4, 1.37 *s*, 3H; OCH_3_: 61.4, 3.77 *s*, 3H, OH at 11: 6.85 *s*; OH at 14: 13.29 *s*.

NMR data of 3-deoxyagastaquinone (8) in CDCl_3_: *δ*_C_ (75.47 MHz, in ppm), δ_H_ (300.13 MHz, in ppm, multiplicity, coupling constants *J* in Hz in brackets): 1: 124.4, 7.72 *d* (10.0), 1H; 2: 131.6, 6.22 *dt* (5.0, 10.0), 1H; 3: 37.6, 2.25 *dd* (2.0, 4.0), 2H; 4: 34.9; 5: 155.7; 6: 120.5, 7.18 *d*, 1H; 7: 161.6; 8: 112.8; 9: 125.2; 10: 127.6; 11: 183.6; 12: 159.8; 13: 137.4; 14: 190.3; 15: 24.1, 3.39 *sept* (7.0), 1H; 16: 20.3, 1.27 *d* (7.0), 3H; 17: 20.3, 1.27 *d* (7.0), 3H; 18: 28.4, 1.28 *s*, 3H; 19: 28.4, 1.28 *s,* 3H; OH: 13.19 *s*; OCH_3_: 60.7, 4.04 *s*.

### 2.5. Cell Culture and PBMC Isolation

The study was performed in accordance with the Declaration of Helsinki. PBMC were isolated from healthy donors who gave written consent that their donated blood may be used for scientific purposes, in case when it was not selected for transfusion. The PBMC were isolated by density gradient centrifugation as described earlier [12]. After isolation, the cells were washed three times with phosphate buffered saline containing 1 *μ*M ethylenediaminetetraacetic acid and subsequently they were maintained in RPMI 1640 (Sigma Aldrich, Vienna, Austria) supplemented with 2 mM L-glutamine, 10% heat-inactivated fetal calf serum (Biochrom, Germany) and 50 *μ*g/mL gentamicin (Lonza-BioWhittaker, USA), at 37°C in a humidified atmosphere containing 5% CO_2_.

The cells were seeded in 48-well plates (1.5 × 10^6^ cells/mL/well) and treated either with increasing concentrations of the extract, the individual fractions or the pure compounds, dissolved in DMSO. The PBMC were either stimulated with 10 *μ*g/mL of the mitogen lectin phytohaemagglutin (PHA) after 30 minutes of treatment, or were left unstimulated. In addition to PHA, the stimulatability of the PBMC was controlled for each experiment by treatment with the lectin concanavalin A (ConA) at a concentration of 10 *μ*g/mL. After 48 h of incubation, cell culture supernatants were collected and stored at −20°C until analysis. In addition, 1-methyl-D-tryptophan (Sigma-Aldrich, Vienna, Austria) was used as a postive control for the suppression of IDO-1 acitivity [25].

### 2.6. Measurements of Tryptophan and Kynurenine Concentrations

Briefly, the HPLC analysis of tryptophan and kynurenine was performed on a ProStar Varian system (USA) using rp-18 columns (Merck, Germany) and acetate buffer as eluent (flow-rate: 0.9 mL/min) according to the protocol described earlier [26, 27]. 3-Nitro-L-tyrosine (Sigma Aldrich, Austria) was used as an internal standard. Kynurenine and tryptophan standards were purchased from Sigma-Aldrich (Austria). Kynurenine and 3-nitro-L-tyrosine were detected by UV-absorbance at 360 nm wavelength (Shimadzu SPD-6A UV detector, Austria), tryptophan was detected by its fluorescence with an excitation wavelength of 286 nm and an emission wavelength of 366 nm (ProStar 360 detector, Varian, USA). The Kyn/Trp was calculated, which is an estimate of IDO-1 activity [12] and expressed in *μ*mol Kyn / mmol Trp. The sensitivity of the measurements was 0.5 *μ*mol/L kynurenine and 0.1 *μ*mol/L tryptophan.

### 2.7. Neopterin Measurements

Neopterin concentrations were measured by ELISA (BRAHMS, Germany), according to the manufacturers' instructions with the detection limit of 2 nmol/L.

### 2.8. IC_50_ Calculations and Statistical Analysis

The half maximal (50% inhibitory) concentration (IC_50_) was calculated by using the CalcuSyn software (Biosoft, UK) according the concept of Chou and Talalay [28]. The Statistical Package for the Social Sciences (SPSS, version 21, Chicago, USA) was used for the analysis of data with Friedman and Wilcoxon signed-rank tests, as not all data showed normal distribution. P-values below 0.05 were considered to indicate significance. Results are expressed as percent of unstimulated and PHA-stimulated control cells and are shown as mean ± standard error of the mean (SEM).

## 3. Results

### 3.1. Tryptophan, Kynurenine, and Neopterin Metabolism in PBMC

**Figure 1:**
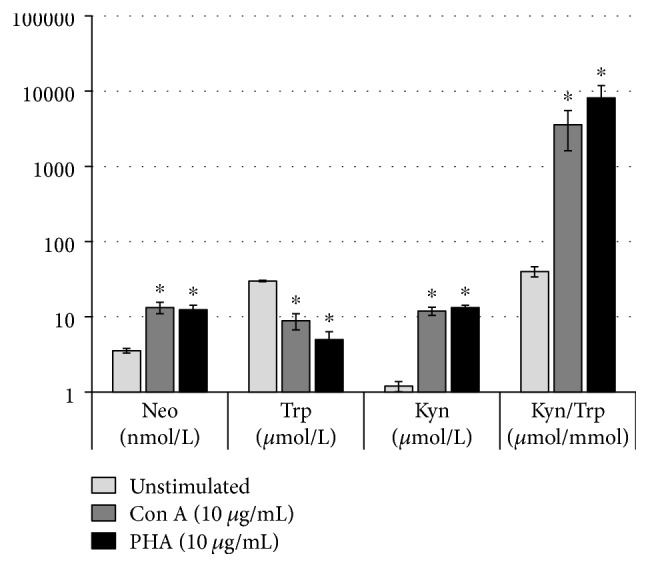
Concentrations of neopterin (Neo), tryptophan (Trp), kynurenine (Kyn) as well as the Kyn/Trp, a measure of IDO-1 activity, in the supernatant of unstimulated PBMC and cells stimulated with 10 *μ*g/mL concanavalin A (Con A) or phytohaemagglutinin (PHA) for 48 h. Results shown are the mean values ± SEM of seven independent experiments run in duplicates (^∗^ *p* < 0.05, compared to unstimulated cells; please note log-scale).

On average (mean ± SEM), concentrations of 29.9 ± 0.8 *μ*mol/L tryptophan and 1.2 ± 0.2 *μ*mol/L kynurenine were measured in the PBMC supernatants from different donors kept in culture for 48 h, resulting in a Kyn/Trp of 40.1 ± 6.1. Neopterin concentrations were 3.5 ± 0.2 nmol/L (*n* = 7). After stimulation with the lectins phytohemagglutinin and concanavalin A, both neotperin, kynurenine and Kyn/Trp levels raised significantly compared to the untreated control, while tryptophan concentrations decreased (Figure 1).

### 3.2. Bioactivity-Guided Fractionation

**Figure 2:**
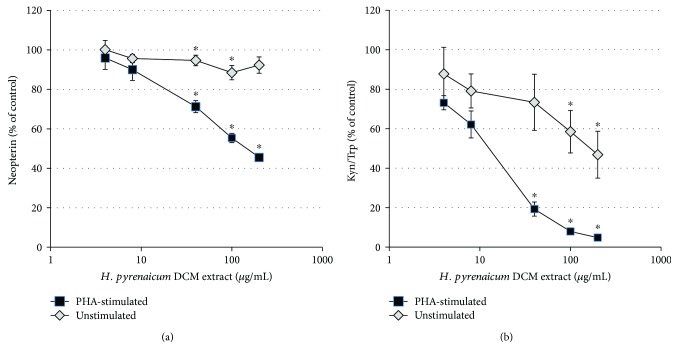
Bioactivity of the *Horminum pyrenaicum* root extract: Unstimulated (grey squares) and phytohemagglutinin (PHA)-stimulated peripheral mononuclear cells (PBMC; black squares) were treated with increasing extract concentrations for 48 h. Neopterin formation (a) and tryptophan breakdown to kynurenine, indicated by the Kyn/Trp (b), was measured in the cell supernatants. Results shown are the mean values ± SEM of three independent experiments run in duplicates. ^∗^ *p* < 0.05 indicates significant differences compared to the respective unstimulated or stimulated control cells (set as 100%).

The DCM extract of *H. pyrenaicum* roots was separated by Sephadex-LH20 column chromatography to obtain 8 fractions (HOR-a1 to HOR-a8). Fractions with similar compound spectra were pooled. Fractions HOR-a3, HOR-a4, HOR-a6, HOR-a7, and HOR-a8 were tested for their immunomodulatory activity in the PBMC cell model to guide the subsequent isolation of bioactive constituents. Several fractions contained bioactive compounds, which dose-dependently suppressed the target pathways (see supplemental figure S1). The half maximal inhibitory concentrations (IC_50_) for neopterin formation and tryptophan breakdown of the most active fractions HOR-a3, HOR-a4, and HOR-a6 can be found in Table 1. The strongest suppressive effect on both mitogen induced pathway was obtained with fraction HOR-a3, for which neopterin formation was suppressed to 50% at a treatment concentration of 63.5 *μ*g/mL and tryptophan breakdown was reduced to 50% with 7.02 *μ*g/mL HOR-a3 (Figure 2).

In unstimulated cells, there was a minor but significant suppressive effect on both pathways upon exposure to HOR-a3, however at higher treatment concentrations only. Due to the stronger relative effect on Kyn/Trp compared to neopterin formation, the tryptophan breakdown pathway was selected as primary decision making-parameter for further subfractionation of the most active fraction HOR-a3.

### 3.3. Isolation of Bioactive Diterpene Quinones

**Figure 3:**
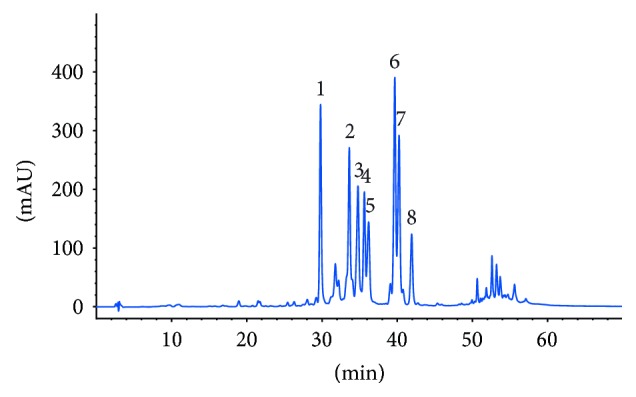
Chromatogram of the HPLC analysis (280 nm) of the bioactive fraction HOR-a3 (1.0 mg/mL) with identified compounds (see Figure 4).

**Figure 4:**
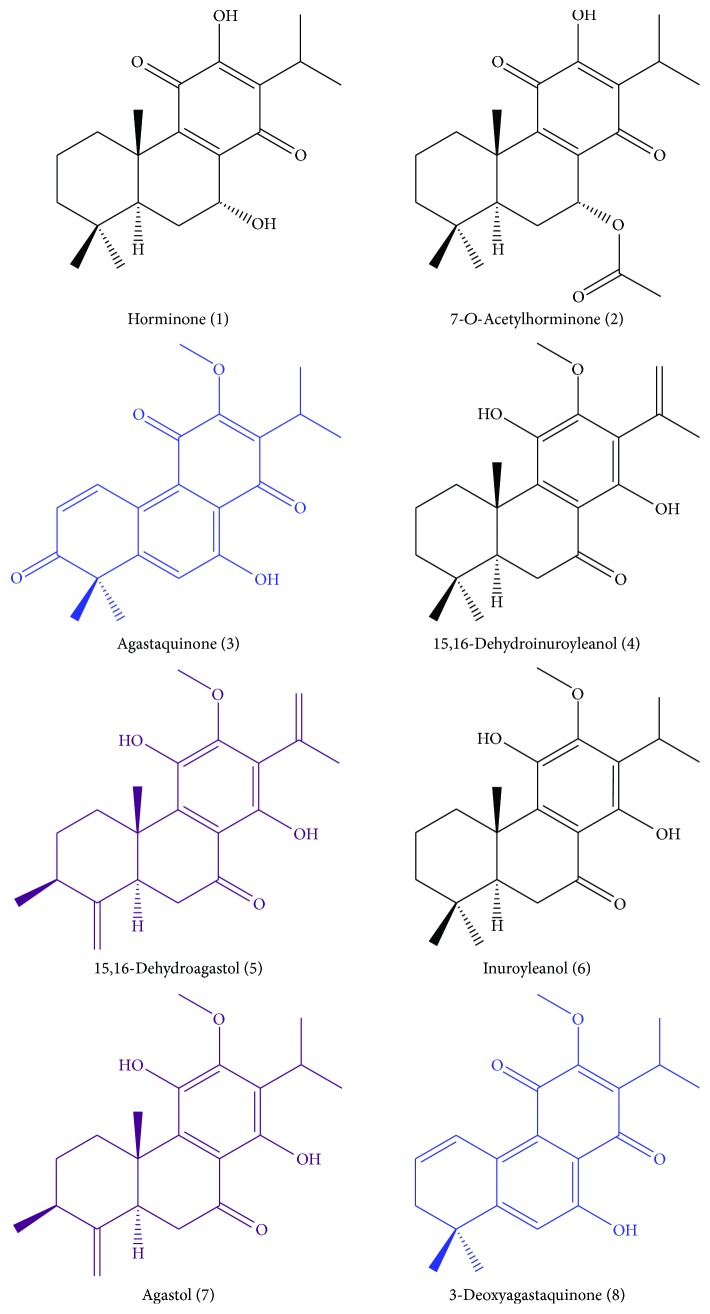
Identified constituents of the roots of *Horminum pyrenaicum*. Black, blue and violet colors represent abietane-diterpene quinones, nor-abietane diterpene quinones, and *abeo* 18 (4 → 3) abietane diterpene quinones, respectively.

Aiming at the isolation of the major compounds present in the bioactive fraction HOR-a3, 10.6 g of the DCM extract of the root material of *H. pyrenaicum* was separated by means of silica gel and Sephadex LH 20 column chromatography, HSCCC, as well as semi-preparative HPLC (Figure 3). This enabled the isolation of six compounds (Figure 4). They were identified by mass spectrometry, 1- and 2-D NMR, and comparison of the spectral data with literature as the diterpene quinones horminone (1) [29, 30], 7-*O*-acetylhorminone (2) [31, 32], agastaquinone (3) [33], inuroyleanol (6) [34, 35], agastol (7) [36, 37], and 3-deoxyagastaquinone (8). Compound 8 was identified as a new natural product.

In addition to these diterpene quinones, of which only compounds 1 and 2 are known constituents of *H. pyrenaicum*, the bioguided separation process yielded fraction HP-r4 comprising two major compounds, which were difficult to separate with the used chromatographic techniques. Therefore, in this case, we did not follow the classical approach, that is, separation and isolation, NMR measurement and identification, and biological testing of the pure compounds, but another approach, that is, testing of the compound mixture and subsequent identification of the compounds by LC-SPE-NMR according to a procedure described by Sturm et al. [24]. One of the compounds comprised in the mixture was identified as 15,16-dehydroagastol (5) [38]. The compound has already been isolated from *Agastache rugosa* [36]. However, it represents a newly identified compound in the species *H. pyrenaicum*. By means of 1D and 2D–NMR-spectroscopy, the second constituent of fraction HP-r4 was identified as 15,16-dehydroinuroyleanol (4), which is a new natural compound (Figure 4).

Consequently, the isolated diterpenchinones 1–3 and 6–8 as well as fraction HP-r4 comprising a mixture of compounds 4 and 5 were tested on their ability to influence Th1-type immune responses.

### 3.4. Anti-Inflammatory Effects of the Isolated Compounds

**Figure 5:**
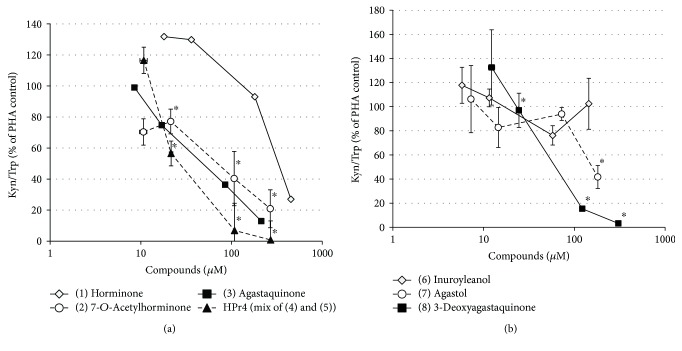
Inhibition of indoleamine 2,3-dioxygenase, as indicated by Kyn/Trp, in mitogen-stimulated PBMC by the identified compounds. PBMC treated with PHA alone were used as a control for each experiment (set as 100%). (A) (1) horminone (*n* = 1), (2) 7-*O*-acetylhorminone (*n* = 3), (3) agastaqiunone (*n* = 2) and HP-r4, which is an equimolar mixture of the compounds (4) 15,16-dehydroinuroyleanol and (5) 15,16-dehydroagastol (the average molecular weight was used for calculations; *n* = 3) and (B) (6) inuroyleanol (*n* = 3), (7) agastol (*n* = 3) and (8) 3-deoxyagastaquinone (*n* = 3). The number of replicates is indicated in brackets behind each compound. It was not possible to repeat the experiments in all cases due to the low amount of substance yield.

All of the known and identified compounds in this study except inuroyleanol (6) were able to influence tryptophan breakdown in stimulated PBMC at micromolar concentrations and in a dose-dependent manner, however, with different intensities (Table 2, Figure 5).

Strongest influences exerted the equimolar compound mixture HP-r4 (1 : 1 mixture of (4) 15,16-dehydroinuroyleanol and (5) 15,16-dehydroagastol), followed by (3) agastaqiunone > (2) 7-*O*-acetylhorminone > (8) 3-deoxyagastaquinone > (7) agastol > (1) horminone as indicated by the IC_50_ values (Table 2, Figure 5).

An inhibition of neopterin formation in PHA-stimulated PBMC was observed for HP-r4 and compounds (1), (2), (8), while compounds (7) showed minor and (3) and (6) showed no dose-dependent effect (data not shown). However, due to sample limitation, neopterin formation could not be determined for compounds (1) and (3) with sufficient repetitions.

## 4. Conclusion

This study reports the isolation process of *H. pyreanicum* compounds in a bio-guided way, where fractions with high immunomodulating properties were used to direct further subfractionation processes. This guided separation of bioactive compounds successfully led to the identification of potent diterpene quinone derivatives, which were able to suppress central Th1-type immunometabolic pathways. A variety of phytochemicals were shown to interfere with IDO-1 activity previously, for example, the antioxidants resveratrol [12], the lavender oil constituents (−)-linalool, (+)-*α*-pinene and (+)-limonene [39], the lignans arctigenin and trachelogenin [40] or the benzylisoquinoline alkaloid berberine [41]. In addition, commonly used anti-inflammatory medications were shown to interfere with the IDO-1 and GTP-CH-I activity, for example, acetylsalicylic acid (aspirin) [42]. These studies further confirm the importance of these pathways to be investigated in mode of action studies of phytochemicals. Furthermore, it should be considered that the suppressive effect can be either indirect by the generation of a more reductive milieu due to the antioxidant nature of several phytochemicals, thereby counteracting T cell activation leading to less IFN-*γ* production [43], or directly as has been shown for example for arctigenin and trachelogenin that interact with the binding site of the enzyme [44]. The investigation of neopterin formation gives a further hint towards the mode of action, as in human macrophages neopterin is produced at the expense of tetrahydrobiopterin (BH4), an essential cofactor for several monooxygenases [45].

Inflammation and immune activation play an important role in the pathogenesis of a variety of diseases and are of importance especially in chronic conditions, when the induction of regulatory T-cells (Treg) counteracts the initial immune activation cascade and contributes to the maintenance of an immunosuppressed state, which is associated with fatal outcome, for example, in patients with cardiovascular disease but also in cancer. This adverse situation is well indicated by increased neopterin and Kyn/Trp levels in patients that are associated with increased mortality [46, 47]. Hence, the regulatory influence of plant compounds could be of importance to normalize the immunological situation in such patients. Central immunological cascades are redox sensitive, thus targeting these processes could be a successful strategy. However, several recent studies point towards the adverse effects mediated by an excessive use of antioxidants. The induced reductive stress is suggested to promote the development of allergies or asthma [48, 49]. This might be considered when botanical extracts or phytochemical antioxidants are used without medical consideration, in excess and/or over long periods.

Bioactivity guided fractionation of the root material of *H. pyrenaicum,* enabled the identification of four abietane-diterpene quinone derivatives as horminone (1), 7-*O*-acetylhorminone (2), inuroyleanol (6) and its 15,16-dehydro-derivative (4). Additionally, the nor-abietanes agastaquinone (3) and 3-deoxyagastaquinone (8), and the *abeo* 18 (4 → 3) abietanes agastol (7) and its 15,16-dehydro-derivative (5) were identified. NMR-data of compounds 1–3 and 5–7 were in agreement with previous reports mentioned above, while compounds (4) and (8) were identified as new natural products. Compounds 1–3 and compounds 6–8 as well as fraction HP-r4 comprising a 1 : 1 mixture of compounds (4) and (5) were tested in the *in vitro* assay for their immune-modulating properties. Except of inuroyleanol (6), all compounds affected mitogen-induced tryptophan breakdown dose-dependently in the tested concentration range; however, the suppressive effect was of different intensity (Table 2, Figure 5).

Interestingly, fraction HP-r4, which comprises compounds (4) and (5), had the strongest effect on human PBMC with an IC_50_ for Kyn/Trp suppression of 25.8 *μ*M. Further investigations on the mode of action of compounds 4 and 5 would be necessary to evaluate their potential as IDO-1 inhibitors.

The here reported properties of the identified compounds clearly support follow-up studies to investigate their effects on further immunological cascades, including neopterin formation. Higher neopterin levels and accelerated tryptophan breakdown is frequently observed in patients with various diseases associated with chronic immune activation [19, 23], thus, the here selected target pathways are of high relevance also for the *in vivo* situation.

The occurrence of so far already known diterpene quinones and the finding of newly identified compounds clearly warrant further investigations of the bioactivities of *H. pyrenaicum* extracts and contained phytochemicals.

Moreover, the results of this study underline that the model system of mitogen induced PBMC using the immunometabolic pathways of tryptophan breakdown and neopterin formation, is a reliable screening approach for a bio-guided fractionation and isolation of immunomodulatory compounds. By using human PBMC from healthy donors, the aspect of crosstalk between different immune competent cells, most importantly between T-cells and monocytes/macrophages is taken into account [12]. Moreover, by using unstimulated and mitogen-stimulated conditions, immune activating and anti-inflammatory properties can be analyzed in parallel. As both neopterin and Kyn/Trp are biomarkers, which have been used for the monitoring of several human diseases, there is a close connection to the *in vivo* situation. Due to the limitations of every *in vitro* system, further research will be needed to extrapolate the findings to the human system. In this regard, the combination of the here reported pathway-focused strategy with an unbiased functional genomics approach could be advantageous in building an interaction model of the compounds of interests with other important immunological pathways, as well as to identify potential unwanted off target effects [50]. In conclusion, the here reported approach justifies future studies for an in depth analysis of the immunomodulatory properties of the reported diterpene quinones.

## Figures and Tables

**Table 1:** Half maximal inhibitory concentration (IC_50_) of *Horminum pyrenaicum* extract sub-fractions (HOR-a3 to HOR-a6) that had the largest effect on neopterin formation and IDO-1 activity, as indicated by the kynurenine to tryptophan ratio in phytohemagglutinin stimulated PBMC. More details are shown in the supplemental figure S1.

	Neopterin formation IC_50_ [*μ*g/mL]	IDO-1 activity IC_50_ [*μ*g/mL]
HOR-a3	63.8	7.02
HOR-a4	121	18.4
HOR-a6	159	34.0

**Table 2:** Half maximal inhibitory concentration (IC_50_) of the isolated compounds on IDO-1 activity, as indicated by the kynurenine to tryptophan ratio in phytohemagglutinin stimulated PBMC, calculated according Chou and Talalay [28] (n.d.: concentration-dependent activity could not be determined in the tested concentration range; cursive: estimated values, only limited repetitions were possible due to sample limitations).

	IDO-1 activity IC_50_ [*μ*M]
(1) Horminone	*346*
(2) 7-*O*-Acetylhorminone	71.7
(3) Agastaqiunone	*46.3*
HP-r4 (1 : 1 mixture of (4) 15,16-dehydroinuroyleanol and (5) 15,16-dehydroagastol)	25.8
(6) Inuroyleanol	n.d.
(7) Agastol	165
(8) 3-Deoxyagastaquinone	79.5
1-Methyl-D-tryptophan (positive control)	9.3

## Supplementary Materials

Supplementary Materials Supplemental figure 1: Bioactivity guided screening of the *Horminum* root extract fractions (HOR) by treatment of unstimulated (full bars) and phytohemagglutinin (PHA)-stimulated peripheral mononuclear cells (PBMC; dashed bars) for 48 h and using neopterin formation (A) and tryptophan breakdown to kynurenine, indicated by the Kyn/Trp (B) as readout. The arrow indicates fraction HOR-a3, which showed the strongest suppressive activity on both pathways. (^∗^ *P* < 0.05, compared to respective unstimulated or stimulated control cells).
