# Supplementary figures and images for: Immunomodulatory Effects of Diterpene Quinone Derivatives from the Roots of Horminum pyrenaicum in Human PBMC
Source: Oxid Med Cell Longev. 2018 Jan 14;2018:2980295. doi: 10.1155/2018/2980295 (PMC5821946; doi:10.1155/2018/2980295)

**Neopterin formation [% of control]**

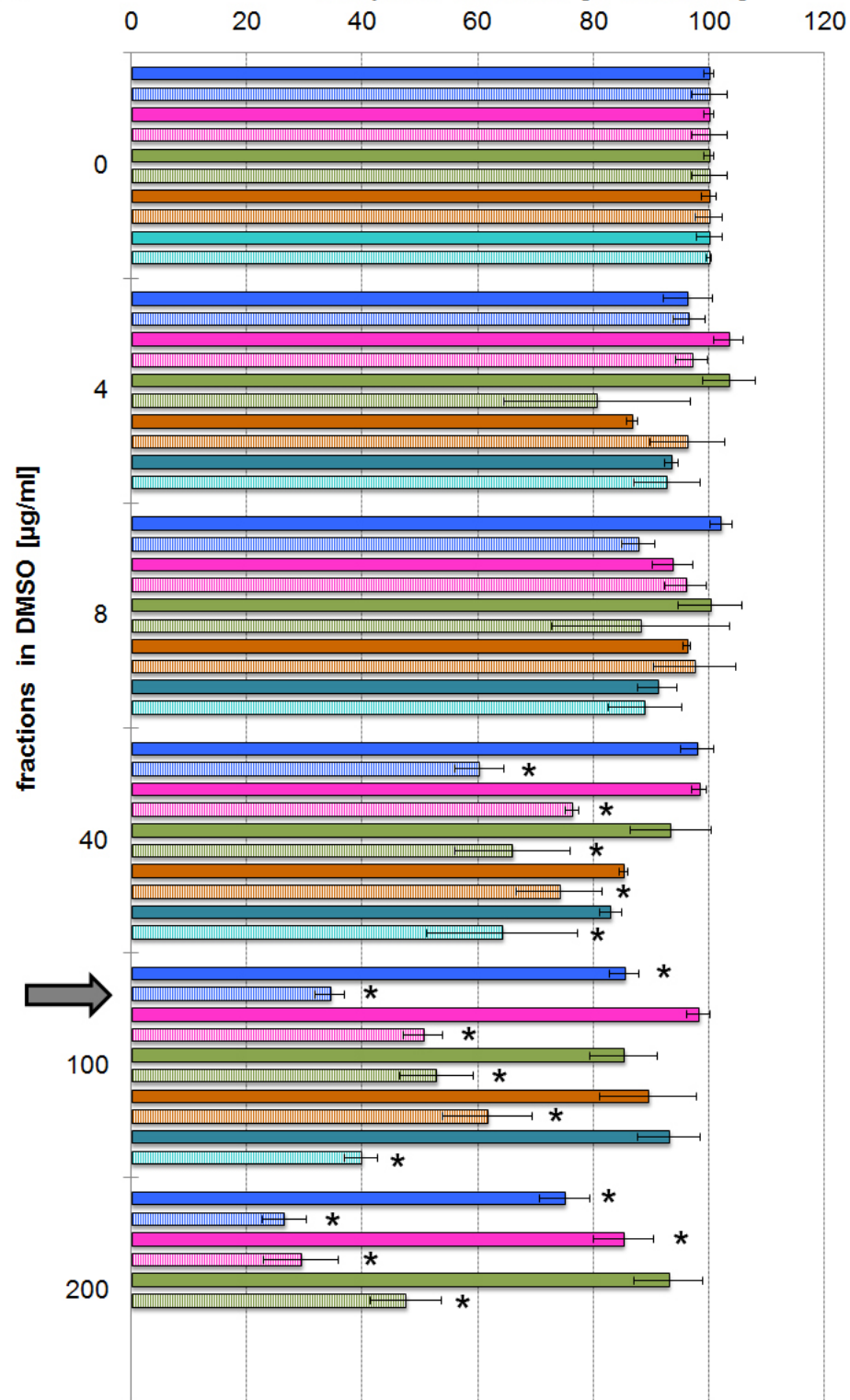

**Kyn/Trp [% of control]**

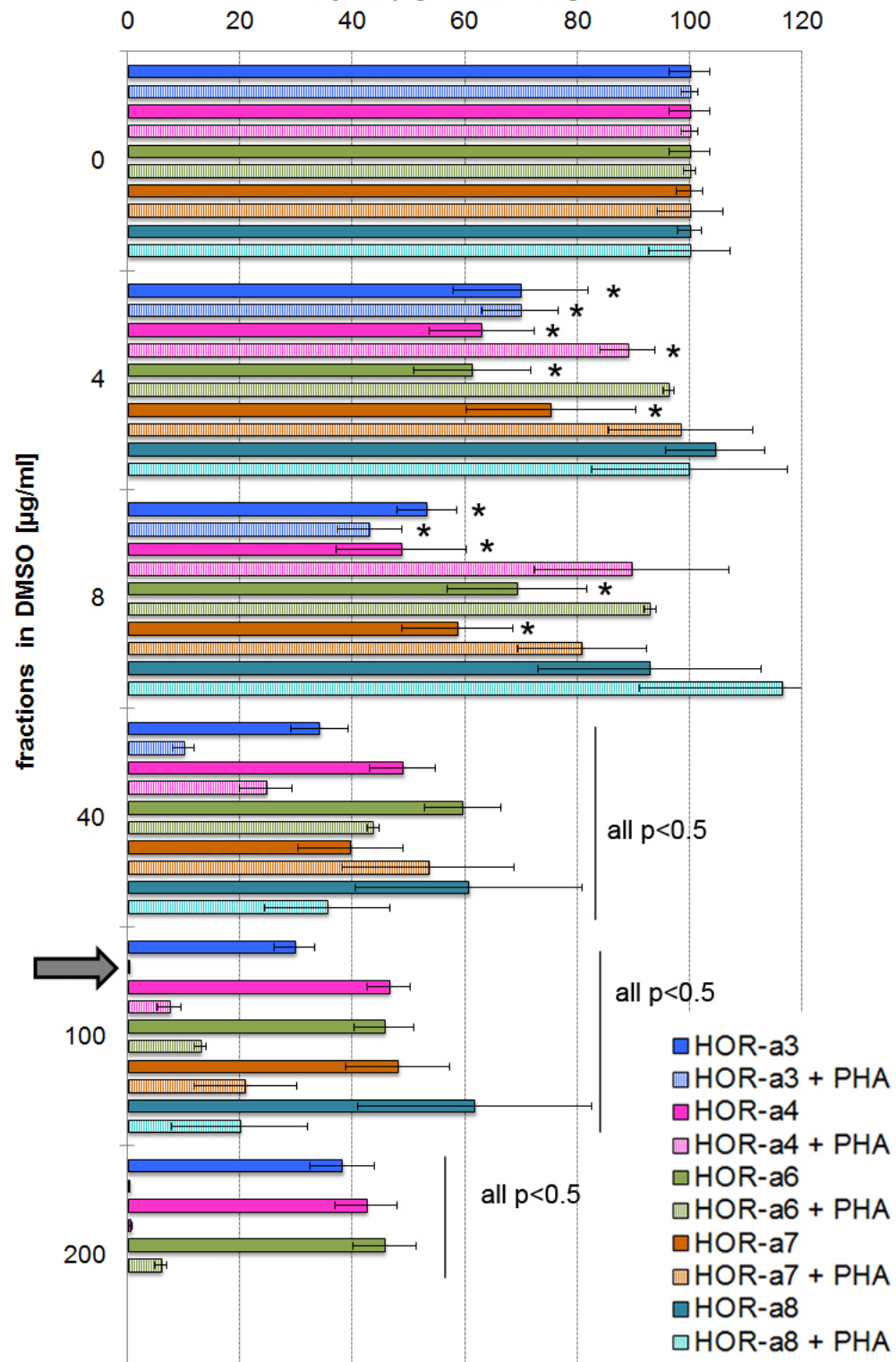

Supplement: Supplementary Materials — Supplemental figure 1: Bioactivity guided screening of the Horminum root extract fractions (HOR) by treatment of unstimulated (full bars) and phytohemagglutinin (PHA)-stimulated peripheral mononuclear cells (PBMC; dashed bars) for 48 h and using neopterin formation (A) and tryptophan breakdown to kynurenine, indicated by the Kyn/Trp (B) as readout. The arrow indicates fraction HOR-a3, which showed the strongest suppressive activity on both pathways. (∗ P < 0.05, compared to respective unstimulated or stimulated control cells). [file 2980295.f1.pdf]
